# Retrospective study on the clinical outcomes and characteristics of acute myeloid leukemia: different outcomes in the same risk group

**DOI:** 10.7717/peerj.20436

**Published:** 2025-12-05

**Authors:** Fanqiao Meng, Maoyuan Xiang, Huan Xie, Yu Liu, Yan Qi, Dongfeng Zeng

**Affiliations:** Department of Hematology, Daping Hospital, The Third Military Medical University, Chongqing, China

**Keywords:** Acute myeloid leukemia, European Leukemia Network, Prognostic stratification, Genetic heterogeneity

## Abstract

**Background:**

Acute myeloid leukemia (AML) remains a prognostically heterogeneous malignancy despite advances in molecular risk stratification. While the 2022 European Leukemia Network (ELN) guidelines refine risk classification, their accuracy in predicting survival outcomes across genetic requires validation.

**Methods:**

We conducted a retrospective analysis of 154 newly diagnosed AML patients at Daping Hospital, integrating next-generation sequencing (NGS)-based genetic profiling, 2022 ELN risk classification, and clinical outcomes.

**Results:**

The most frequent mutations were *FLT3* (26.6%), *DNMT3A* (21.4%), *NPM1* (18.2%), *CEBPA* (17.5%), and *TET2* (15.6%). Median overall survival (OS) and progression-free survival (PFS) were 22.9 months and 14.1 months, respectively. Hematopoietic stem cell transplantation (HSCT), female sex, age < 60 years, and normal karyotype emerged as favorable prognostic factors. No significant differences were observed between allogeneic (allo-HSCT) and autologous HSCT (ASCT). Idarubicin, cytarabine, etoposide (IA ± E) chemotherapy yielded superior survival, while azacitidine+venetoclax (AZA+VEN) regimens underperformed. Conversely, *TP53* and *KIT* mutations correlated with inferior survival, while *NPM1, CEBPA* mutations predicted longer survival. Notably, significant survival heterogeneity existed within 2022 ELN risk groups, particularly among patients with *FLT3*, *CEBPA*, or *TET2* mutations.

**Conclusions:**

The ELN risk classification demonstrate limitations in prognostication, particularly for patients with *FLT3*, *CEBPA*, or *TET2* mutations. Our findings highlight the necessity for refined risk models incorporating additional molecular markers (*KIT*) and mutation interactions to enhance personalized prognostication. Gene coexistence is also a factor that needs to be considered when determining patient prognosis.

**Trial registration:**

The study was registered on the Chinese clinical trial registry (ChiCTR) platform (No. ChiCTR2500096484).

## Introduction

Acute myeloid leukemia (AML) is a molecularly heterogeneous clonal disorder characterized by dysregulated myeloid differentiation and dismal survival rates in high-risk subgroups (Kantarjian et al., 2021). Contemporary risk stratification, guided by the National Comprehensive Cancer Network (NCCN) and European Leukemia Network (ELN) criteria, integrates cytogenetic and molecular abnormalities to inform therapeutic decisions (Lachowiez et al., 2023; Pollyea et al., 2023). The prognostic significance of these classifications underscores their critical role in optimizing AML management.

The 2022 ELN guidelines represent a paradigm shift in AML risk classification, revising key criteria from the 2017 version. Notably, FLT3-ITD mutations—previously stratified based on allelic ratio—are now uniformly classified as intermediate-risk when occurring without adverse genetic features. This reclassification primarily addresses methodological challenges in standardizing allele ratio assessment, the emerging role of measurable residual disease (MRD) dynamics in *NPM1*-mutated AML, and the therapeutic impact of targeted kinase inhibitors (Falini & Dillon, 2024). In addition, FLT3-ITD MRD analysis, which is based on next-generation sequencing (NGS), has improved the ability to predict survival outcomes (Ediriwickrema, Gentles & Majeti, 2023; Peroni et al., 2023). Mutations in AML with mutations in nine myelodysplasia-related (AML-MR) genes (*ASXL1, BCOR, EZH2, RUNX1, SF3B1, SRSF2, STAG2, U2AF1*, or *ZRSR2*) are designated as high-risk, independent of other cytogenetic abnormalities. *CEBPA* biallelic or monoallelic mutations within the bZIP domain are classified as favorable, irrespective of their allelic status. Additionally, hyperdiploid karyotypes with multiple trisomies are no longer considered complex or high-risk, reflecting advances in understanding their prognostic implications (Bouligny, Maher & Grant, 2023; Lachowiez et al., 2023).

Despite these updates, the 2022 ELN framework faces unresolved limitations (Lo et al., 2023). The combination of MRD and risk stratification can guide the treatment of AML (Cloos, Ngai & Heuser, 2023). In addition, studies have shown that age should also be considered in the risk stratification of AML patients (Pogosova-Agadjanyan et al., 2020). Clinical and molecular heterogeneity persist within risk groups, particularly among patients with “normal” karyotypes or co-occurring mutations (Dohner, Weisdorf & Bloomfield, 2015; Mrozek et al., 2012). For instance, patients classified as favorable or intermediate-risk by ELN 2022 exhibit marked survival variability, underscoring the inadequacy of current stratification criteria. Furthermore, *NPM1* and *CEBPA* mutated AML, while generally sensitive to induction chemotherapy, demonstrate inconsistent long-term outcomes due to high relapse rates (Shi et al., 2024; Su et al., 2022; Suzuki et al., 2005; Wang et al., 2020). The integration of novel therapies—such as venetoclax (VEN)-based regimens and hematopoietic stem cell transplantation (HSCT)—has improved survival even in historically high-risk cohorts, yet these advances challenge traditional risk models (Socie, 2022). AML-RC are classified into an adverse risk group, but the prognosis for these patients varies.

However, persistent outcome disparities within risk strata highlight unmet needs in prognostic precision. This study conducts a comprehensive analysis of clinical characteristics and survival outcomes in newly diagnosed AML patients, addressing unresolved controversies in current risk stratification.

## Materials & Methods

### Study design and participants

The incidence rate of AML in China is approximately 1.62 per 100,000 people. The data is derived from the “Chinese Disease Database”. we chose an incidence rate of 2 per 100,000 for calculating the required sample size. The sample size calculation formula is as follows: $ \frac{{z}_{ \frac{a}{2} }^{2}P \left( 1-P \right) }{{\delta }^{2}} ,\alpha $ is set at 0.05 (generally, a difference less than 0.05 is considered statistically significant), Z*α*/2 = 1.96, P is 0.002%, and *δ* is 0.01. The calculated sample size is 77 cases. We collected 154 cases, and the sample size meets the requirements of this retrospective study. In this study, we used the FAB classification for AML. We conducted a retrospective cohort study of 154 consecutive AML patients (excluding AML-M3) diagnosed between January 2018 and February 2025 in the Department of Hematology, Daping Hospital, Third Military Medical University (Army Medical University). Diagnosis followed 2016 World Health Organization (WHO) criteria, with risk stratification per ELN 2022. Clinical information was retrieved from the electronic medical records.

### Endpoints and assessments

Overall survival (OS) was defined as the time from the first day of diagnosis to death or the last follow-up. Progression-free survival (PFS) was measured from the start of therapy to the date of treatment failure, progression, or death from any cause at the last follow-up.

### NGS and karyotype analysis

Based on international authoritative guidelines (such as ELN, NCCN) and the Leukemia Expert Committee of the Chinese Society of Clinical Oncology (CSCO), the laboratory of our department has detected 56 genes closely related to myeloid tumors for AML diagnosis. The clinical grading interpretation of the filtered gene mutation sites is in accordance with the Li et al. (2017). Using public databases such as dbSNP, 1,000 Genomes, gnomAD, COSMIC, ClinVar, and laboratory self-built hematological tumor database, the variant data is filtered, screened, and graded to obtain the analysis results. Bone marrow (BM) samples underwent NGS targeting 56 myeloid-related genes, as showed in Table S1 (average depth: 2,000 ×). These genes can be divided into eight categories according to their functions (Table S2). Cytogenetics plays a significant role in AML, influencing aspects such as onset, risk stratification, treatment strategy selection, and survival prognosis. The ELN score classifies AML patients into low-risk, intermediate-risk, and high-risk groups by integrating information on gene mutations, chromosomal abnormalities, and molecular markers, thereby optimizing treatment strategies. G-banding analysis of metaphase spreads (≥20 cells) classified karyotypes per the International System for Human Cytogenetic Nomenclature (ISCN) (Simons, Shaffer & Hastings, 2013).

### Study approval and informed consent

The study protocol was conducted by the principles of the Declaration of Helsinki and approved by the Ethics Committee Review Board of the Third Hospital of Army Medical University (2025-09). The study was registered on the Chinese clinical trial registry (ChiCTR) platform (No. ChiCTR2500096484). Written informed consent was obtained from all participants or their legally authorized representatives prior to enrollment in the study.

### Statistical analysis

Continuous variables were reported as medians (ranges) and categorical variables as frequencies (%). Survival analyses utilized Kaplan–Meier estimates with log-rank testing. Cox proportional hazards models identified prognostic factors (two-sided *P* < 0.05). Analyses were performed in R v4.2.2 and GraphPad Prism 9.0.

## Results

### Baseline patient characteristics

The baseline characteristics of the patients are summarized in Table 1. Of 154 patients (median age: 52 years, range: 15–86), 44.8% were male. Common AML subtypes were AML-M2 (48.1%) and AML-M5 (31.8%). There were three patients with mixed-phenotype leukemia (MPAL) and seven patients with AML-RC. Median white blood cell (WBC) count, hemoglobin, and platelets were 8.79  ×  10^9^/L,78.0 g/L, and 35.0  ×  10^9^/L, respectively. Fifty-four percent had a normal karyotype. A total of 67 patients underwent HSCT, with 54 receiving allogeneic HSCT (allo-HSCT) and 13 receiving autologous HSCT (ASCT). Induction therapies included 7+3 regimens (40.9%), azacytidine+venetoclax (AZA+VEN) (15.6%), and others.

**Table 1:** Baseline characteristics of AML patients at enrollment.

**Characteristics**	**Value**
**Male, n (%)**	69 (44.8%)
**Age, M (range) years**	52 (15–86)
Age ≥ 60 years	45 (29.2%)
**Laboratory, median (range)**	
WBC, M (range) ×10^9^/L	8.79 (0.62–317.53)
Hb, M (range) × g/L	78 (27–148)
PLT, M (range) ×10^9^/L	35 (4–366)
BM blast (%), median (range)	59.5 (17.50–98.50)
PB blast (%), median (range)	42.0 (0.0–97.0)
LDH, median (range) U/L	371 (100.4–5,077.9)
**FAB classification, n (%)**	
M0	0 (0.0%)
M1	10 (6.5%)
M2	74 (48.1%)
M4	10 (6.5%)
M5	49 (31.8%)
M6	1 (0.6%)
M7	0 (0.0%)
MPAL	3 (1.9%)
AML-MR	7 (4.5%)
**Induction chemotherapy**	
IA	16 (10.4%)
IAE	8 (5.2%)
DA	47 (30.5%)
DAE	30 (19.5%)
CAG	5 (3.2%)
HA	7 (4.5%)
AZA+VEN	24 (15.6%)
Others	17 (11.0%)
**Cytogenetic karyotype**	
Normal, n (%)	84 (54.5%)
HSCT	67 (43.5%)
Allo-HSCT	54 (35.1%)
ASCT	13 (8.4%)

**Notes.**

WBC white blood cell HB hemoglobin PLT hemoglobin BM bone marrow PB peripheral blood BM% proportion of BM blasts PB% proportion of PB blasts LDH lactate dehydrogenase IA idarubicin, cytarabine IAE idarubicin, cytarabine, etoposide DA daunorubicin, cytarabine DAE daunorubicin, cytarabine, etoposide CAG cytarabine, aclarubicin and granulocyte colony-stimulating factor HA homoharringtonine, cytarabine AZA azacitidine VEN venetoclax HSCT hematopoietic stem cell transplantation Allo-HSCT allogeneic hematopoietic stem cell transplantation ASCT autologous hematopoietic stem cell transplantation

### Cytogenetic analysis

Cytogenetic analysis was available for 151 patients. Normal karyotypes were observed in 84 patients (54.5%), and chromosome karyotype analysis failed in three patients (1.9%). Solitary abnormalities were detected in 40 (26%) patients, and two abnormalities were detected in 12 (7.8%) patients. Complex karyotypes were observed in 22 (14.3%) patients, and karyotypes with ≥ 3 abnormalities were detected in 1 (0.6%) patient. According to 2022 ELN (only chromosome analysis), 5.8%, 76.6%, and 15.6% of patients are categorized as favorable, intermediate, and adverse risk, respectively. The cytogenetic data are summarized in Table 2.

**Table 2:** Cytogenetic analysis in AML patients.

**Karyotype at diagnosis**	**n (%)**
Normal karyotype	84 (54.5%)
inv (16)	4 (2.6)
t (16; 16)	1 (0.6%)
t (8; 21)	13
Single abnormality	40 (26.0%)
Two abnormalities	12 (7.8%)
≥ 3 abnormalities	1 (0.6%)
Complex karyotype	22 (14.3%)
ND	3 (1.9%)
Cytogenetic risk (2022 ELN)	
Favorable	9 (5.8%)
Intermediate	118 (76.6%)
Poor	24 (15.6%)

**Notes.**

ND not detected

### Genomic profiling results

**Figure 1:**
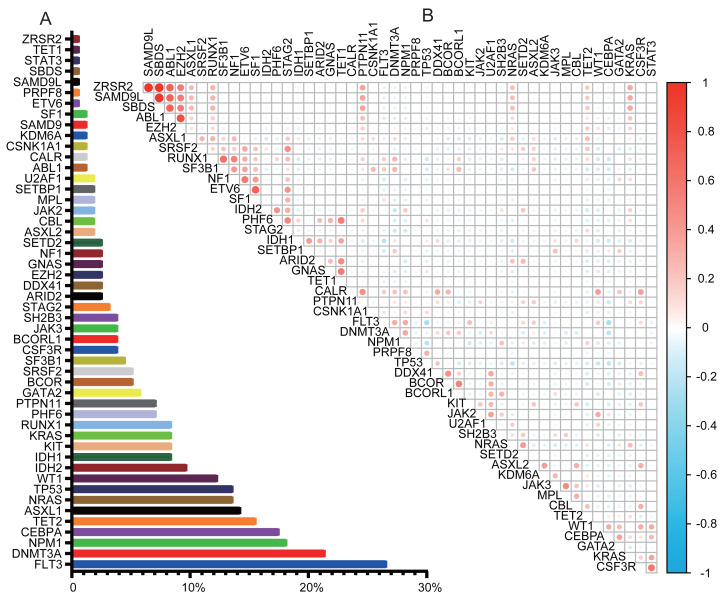
Gene mutation landscape in AML. (A) Bar graph showing the frequency of mutations in AML. (B) Co-mutations and exclusivity patterns of AML. Blue: Exclusivity relationship; Red: co-occurring relationship. Higher color intensity indicates a stronger association.

NGS was performed on 154 patients, covering 56 genes. NGS detected mutations in 94.2% of patients. Complex variations involving more than three gene mutations were observed in 56.5% (87/154). The genetic landscapes of these patients are shown in Fig. S1. Of the 56 genes analyzed, six were not detected: *CDC25C, ETNK1, GNB1, PIGA, PPM1D*, and *UBA1*. The most frequently mutated genes were *FLT3* (26.62%), *DNMT3A* (21.43%), *NPM1* (18.18%), *CEBPA* (17.53%), *TET2* (15.58%), *ASXL1* (14.29%), *TP53* (13.64%), *NRAS* (13.64%), and *WT1* (12.34%), as illustrated in Fig. 1A. The comutation and exlusivity patterns were examined (Fig. 1B). *FLT3* co-occurred with *NPM1* and *DNMT3A* but was mutually exclusive with *TP53* and *CEBPA*. *TP53* mutations were associated with *DDX41* but excluded *NRAS*, *WT1*, *FLT3*, and *NPM1*. *CEBPA* was strongly associated with *WT1*, but mutually exclusive to both *TP53* and *FLT3* mutations.

### ELN risk reclassification

Risk stratification according to both the 2017 and 2022 ELN is shown in Fig. S2. Compared to 2017 ELN, the 2022 update reduced the favorable group (22.7% to 18.1%) and increased the adverse group (42.8% to 47.4%), with minimal change in the intermediate group. Twenty-four patients (15.6%) had discordant risk classifications between the two systems.

### Survival

**Figure 2:**
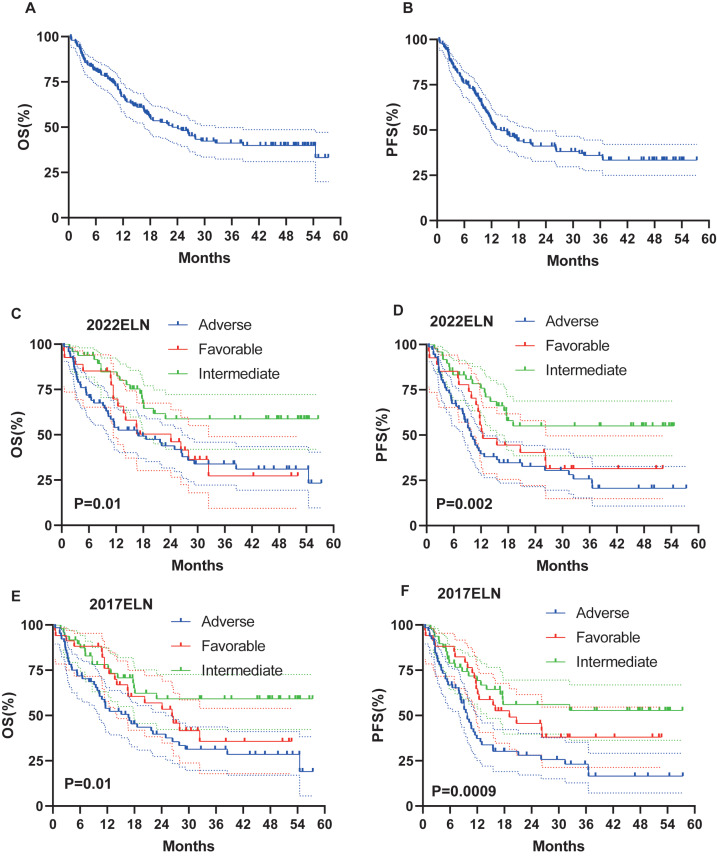
OS and PFS of all patients, based on 2017 and 2022 ELN classifications. (A–B) OS and PFS of all patients. (C–D) OS and PFS based on ELN 2022 criteria in all patients. (E–F) OS and PFS based on ELN 2017 criteria in all patients.

Survival data were available for 146 patients. Median follow-up was 16.8 months (range: 0.5–57.4). At the last follow-up, a total of 79 (54.1%) patients had died. Median OS and PFS were 22.9 months and 14.07 months, respectively. Three-year OS and PFS rates were 41% and 36% (Figs. 2A–2B).

### Impact of ELN stratification on survival

On the basis of the 2022 ELN and 2017 ELN risk stratification models, the median OS (16.9 months *vs* 16.67 months) and PFS (9.8 months *vs* 9.8 months) results in the intermediate and adverse risk groups are basically the same, whereas, for the favorable risk groups, the median OS (24.13 months *vs* 26.33 months) and PFS (12.4 months *vs* 19.1 months) of the 2022 ELN stratification model are both shorter than those of the 2017 ELN stratification model (Figs. 2C–2F).

### Survival with HSCT

**Figure 3:**
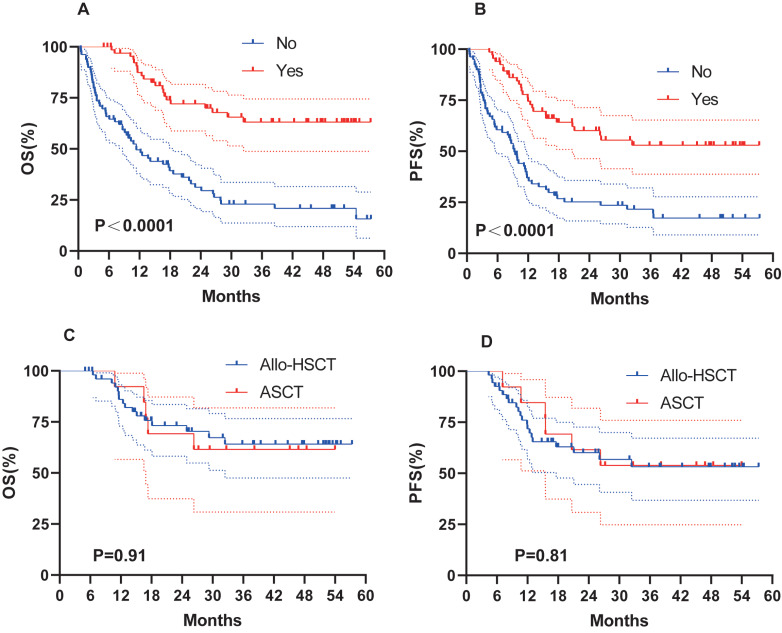
Survival analysis of AML patients with HSCT. OS (A) and PFS (B) of AML patients treated with HSCT. (C–D) OS and PFS of AML patients treated with Allo-HSCT and ASCT.

Of the 146 patients under follow-up, 67 underwent HSCT, which significantly improved survival outcomes. HSCT significantly improved OS (not reached *vs.* 11.4 months, *P* < 0.0001; Fig. 3A) and PFS (not reached *vs.* 9.3 months, *P* < 0.0001; Fig. 3B). Our transplant group consisted of 54 allo-HSCT and 13 ASCT. To determine whether the type of transplantation influenced survival, we conducted a further analysis. No significant difference was observed between allo-HSCT and ASCT (Figs. 3C–3D).

### Clinical factors and prognosis

**Figure 4:**
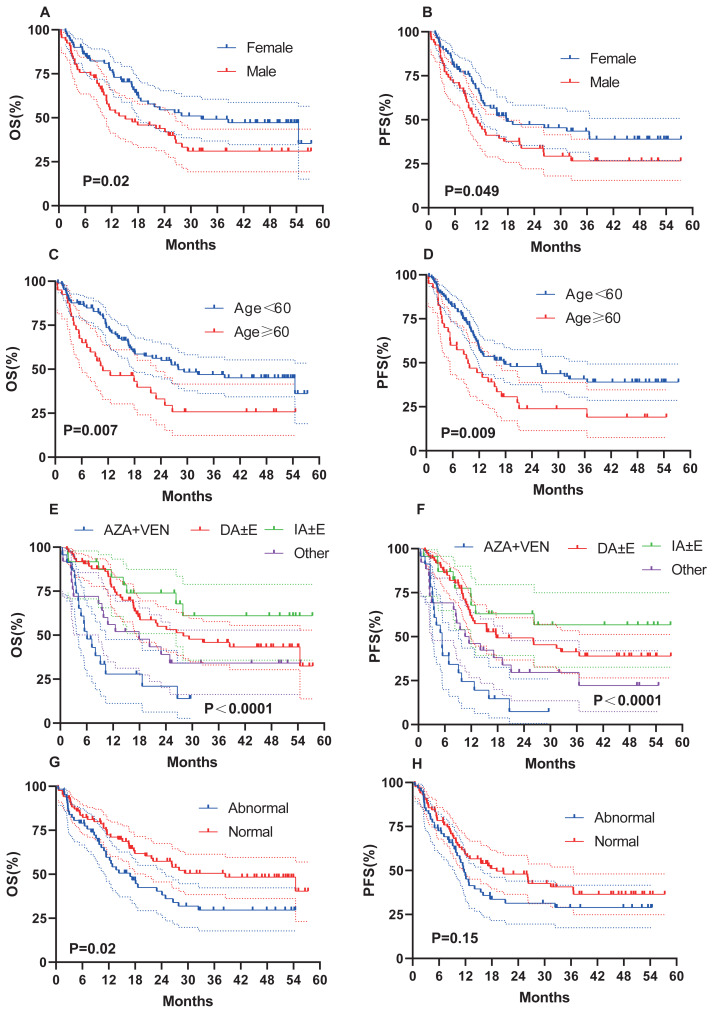
OS and PFS of AML patients based on clinical features. (A–B) OS and PFS based on gender. (C–D) OS and PFS based on age. (E–F) OS and PFS based on chemotherapy. (G–H) OS and PFS based on karyotypes.

**Figure 5:**
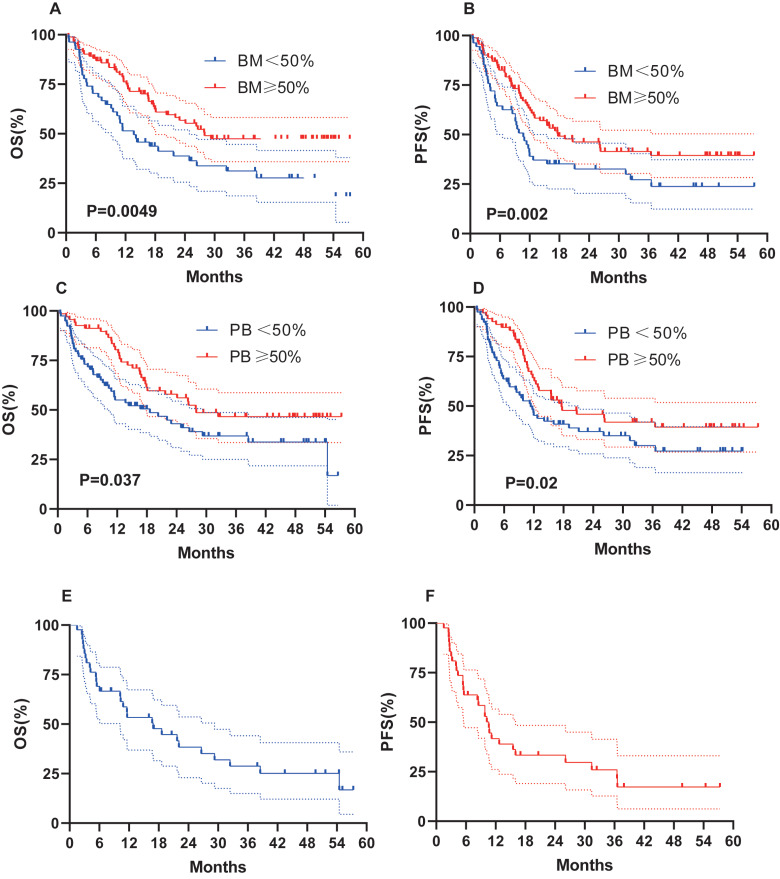
OS and PFS of patients based on BM%, PB% and patients with AML-MR. (A–B) OS and PFS based on BM%. (C–D) OS and PFS based on PB%. (E–F) OS and PFS based on AML-MR.

We evaluated whether other risk factors, including age, sex, induction chemotherapy regimen, chromosome karyotype, proportion of BM blasts % (BM%), and proportion of peripheral blood (PB) blasts % (PB%) at initial diagnosis, might affect survival outcomes (Fig. 4). Female sex (OS, 32.4 months *vs* 15.1 months, *P* = 0.02; PFS, 17.7 months *vs* 10.9 months, *P* = 0.049; Figs. 4A–4B), age < 60 years (OS, 27.9 months *vs* 10.8 months, *P* = 0.007; PFS, 17.7 months *vs* 9.5 months, *P* = 0.009; Figs. 4C–4D), and a normal karyotype (OS, 38.5 months *vs* 16.9 months, *P* = 0.02; PFS, 19.1 months *vs* 11.7 months, *P* = 0.15; Figs. 4G–4H) were associated with longer OS and PFS. However, chromosome karyotype seemed to have little effect on PFS (*P* > 0.05). The chemotherapy regimen was also an important factor, and the IA ± E had longest OS and PFS, the AZA+VEN had the shortest survival (Figs. 4E–4F). BM% ≥ 50% and PB% ≥ 50% were associated with longer OS and PFS (Figs. 5A–5D).

### Outcomes of patients with AML-RC

Forty-two patients (27.3%) were AML-RC, and 29 patients of these (69.5%) died by the time of follow-up. Median OS and PFS were 16.9 months and 10.6 months, respectively, indicating a prognosis comparable to *ASXL1*-mutated AML (Figs. 5E–5F).

### Prognostic importance of genetic variables for survival

Survival outcomes associated with gene mutations were evaluated (Table 3). TP53 mutations conferred the worst prognosis (median OS: 9.1 months; PFS: 5.3 months), followed by *KIT* (OS: 13.7 months; PFS: 9.3 months). *NPM1* and *CEBPA* mutations predicted better outcomes (*NPM1*: OS 38.5 months 26.2 months; *CEBPA*: OS 27.9 months 17.1 months).

**Table 3:** Comparison of survival outcomes in patients with AML based on the presence of gene mutations.

**Gene**	**N**	**Median OS (months)**	**Median PFS (months)**
*FLT3*	39	27.9	17.1
*DNMT3A*	31	17.9	14.1
*NPM1*	30	38.5	26.2
*CEBPA*	26	NR	20.7
*TET2*	21	22.1	26.1
*ASXL1*	20	16.7	10.6
*TP53*	20	9.1	5.3
*NRAS*	19	26.3	17.6
*WT1*	19	NR	10.6
*IDH1*	13	NR	NR
*IDH2*	14	NR	NR
*KIT*	13	13.7	9.3

**Notes.**

OS overall survival PFS progression-free survival NR not reach

### Univariate and multivariate analyses of OS and EFS

The results of the univariate and multivariate analyses of OS and PFS are shown in Figs. S3–S6 and Table 4. In the univariate analysis, age, chemotherapy, HSCT, BM%, and PB% were associated with OS and PFS, while sex and karyotype were also associated with OS but not with PFS. In the multivariate analysis, age, chemotherapy, and HSCT were associated with OS and PFS, whereas BM% and karyotype were also associated with OS but not PFS. Age, chemotherapy (IA ± E), and HSCT were independent predictors of OS and PFS. Notably, age demonstrated conflicting results in univariate *vs.* multivariate analyses, suggesting confounding by other factors.

**Table 4:** Univariate and multivariate Cox-proportional hazard regression analyses for OS and PFS.

**Variables**	**OS**		**PFS**	
	**HR (95% CI)**	***P* value**	**HR (95% CI)**	***P* value**
Univariate analysis				
Male gender	1.63 (1.05–2.54)	0.031	1.52 (1.00–2.32)	0.051
Age ≥ 60	1.88 (1.18–3.00)	0.008	1.79 (1.15–2.79)	0.01
HB ≥ 70	0.95 (0.60–1.48)	0.808	0.91 (0.59–1.41)	0.684
PLT ≥ 30	0.95 (0.61–1.48)	0.818	0.77 (0.51–1.17)	0.226
Normal karyotype	0.61 (0.39–0.95)	0.028	0.73 (0.48–1.12)	0.152
Normal LDH	0.64 (0.38–1.06)	0.083	0.62 (0.38–1.00)	0.051
BM (%) ≥50	0.53 (0.34–0.83)	0.005	0.61 (0.40–0.94)	0.023
PB (%) ≥50	0.59 (0.38–0.93)	0.023	0.64 (0.42–0.98)	0.039
Chemotherapy				
DA ± E	0.31 (0.17–0.54)	<0.0001	0.29 (0.16–0.50)	<0.0001
IA ± E	0.16 (0.07–0.40)	<0.0001	0.19 (0.09–0.43)	<0.0001
Other	0.51 (0.26–1.01)	0.052	0.49 (0.26–0.92)	0.028
HSCT	0.26 (0.16–0.43)	<0.0001	0.32 (0.21–0.51)	<0.0001
Multivariate analysis				
Age ≥ 60	0.29 (0.12–0.70)	0.006	0.29 (0.12–0.66)	0.003
Normal karyotype	0.60 (0.37–0.97)	0.036	0.75 (0.48–1.18)	0.212
BM (%) ≥50	0.52 (0.30–0.90)	0.018	0.64 (0.38–1.08)	0.096
PB (%) ≥50	1.06 (0.59–1.93)	0.837	1.03 (0.58–1.84)	0.919
Chemotherapy				
DA ± E	0.19 (0.06–0.58)	0.004	0.16 (0.05–0.44)	<0.0001
IA ± E	0.14 (0.04–0.52)	0.004	0.14 (0.04–0.48)	0.002
Other	0.58 (0.25–1.38)	0.219	0.48 (0.21–1.09)	0.081
HSCT	0.29 (0.17–0.49)	<0.0001	0.36 (0.22–0.60)	<0.0001

### Discordant outcomes within ELN risk groups

Patients in the 2022 ELN favorable/intermediate (Group A) and adverse (Group B) groups exhibited divergent outcomes. With an OS of 18 months as the criterion, a subgroup analysis was urgently conducted: A (OS > 18 months), A (OS < 18 months), B (OS > 18 months), and B (OS < 18 months). Table S3 shows the patient-related variables in the groups of patients. Subgroup analysis revealed that *FLT3, DNMT3A, NPM1, KIT, CEBPA*, and *TET2* mutations in Group A (Table S4) and *ASXL1, CEBPA, TET2, WT1, NRAS*, and *FLT3* in Group B (Table S5) influenced prognosis. These findings highlight the prognostic impact of specific mutations beyond 2022 ELN classifications.

## Discussion

AML prognosis is influenced by genetic and clinical factors, necessitating individualized risk stratification (Venugopal & Sekeres, 2024). To our knowledge, our retrospective study is leveraging real-world data to evaluate the prognostic utility of NGS-based risk classification under the 2022 ELN framework. We investigated the prognostic implications of genetic mutations, clinical features, and treatment modalities under the 2022 ELN framework. Notably, while the 2022 ELN guidelines enhance the accuracy of intermediate/adverse risk stratification, they exhibit limitations in capturing intragroup variability, particularly among patients harboring *FLT3*, *CEBPA*, or *TET2* mutations. Mutations in *TP53* and *KIT* emerged as strong predictors of dismal outcomes, underscoring their necessity for inclusion in future prognostic models. The 2022 ELN framework demonstrated superior discriminatory power for OS and PFS compared to 2017 ELN, particularly in refining adverse-risk classification (Dohner et al., 2022).

HSCT confers a survival advantage in AML patients, with no significant differences observed between allo-HSCT and ASCT (Chen et al., 2023). At present, there are still controversies regarding the selection criteria and therapeutic efficacy of allo-HSCT and ASCT in AML. Patients with intermediate to high risk may be more likely to benefit from allo-HSCT. However, some studies have found that for low-risk patients such as Core-binding factor acute myeloid leukemia (CBF-AML), the efficacy of ASCT is superior to that of allo-HSCT (Al Hamed et al., 2024). Due to the limitations of our sample size, this conclusion still requires further verification. Our study identified female sex, age < 60 years, and normal karyotype as independent predictors of prolonged OS and PFS. Notably, conflicting results emerged between univariate and multivariate analyses for age, suggesting that its prognostic impact may be modulated by co-factors such as mutation profiles or treatment intensity. Although age remained a significant predictor in the univariate analysis, its marginal effect in multivariate modeling likely reflects the limited representation of elderly patients (15.6%) in our cohort. In addition, our study revealed that BM% ≥50% and PB% ≥50% are not factors for poor prognosis; in contrast, they are factors for good prognosis, which is not quite what we thought. This result needs to be further verified.

According to the Chinese guidelines for diagnosis and treatment of adult AML, for all AML patients who are eligible to participate in clinical studies, it is recommended that they primarily participate in clinical studies. For patients who are suitable for intensive treatment, the IA or DA regimens are the mainstream options. For those who are not suitable for intensive treatment or are elderly and frail, the AZA + VEN regimen is the mainstream option (Leukemia Lymphoma Group CSoHCMA, 2023). There are no uniform OS or PFS values applicable to all Chinese AML patients. This is usually significantly influenced by factors such as age, genetic risk, physical condition and treatment tolerance. One retrospective, real-world study demonstrated that patients with AML treated with VEN+AZA at a single centre had a median OS of 11.93 months (Bai et al., 2025). Our study revealed that chemotherapy prolonged survival, with the IA ± E regimen resulting in the longest OS and PFS and AZA+VEN resulting in the shortest OS and PFS, which is consistent with the findings of one study (Bell et al., 2019). The reason for this result may be that AZA+VEN is mainly used for elderly patients, those with multiple comorbidities or poor physical condition, or those who cannot tolerate intensive chemotherapy. These patients have a poorer prognosis due to their underlying diseases and physical conditions, which limit the intensity and efficacy of the treatment. The AZA+VEN regimen is a low-intensity chemotherapy approach, which is unable to completely eliminate leukemia cells, has a high recurrence rate, and a low long-term survival rate. In addition, HSCT is also an important factor in prolonging the survival prognosis of patients.The 2022 ELN and 2017 ELN are not suitable for elderly patients receiving nonintensive therapy, especially AZA+VEN (Jahn et al., 2023). These patients can be referred to 2022 ELN patients receiving low-intensity therapy (Dohner et al., 2024a). Our results are consistent with reports that the IA regimen prolongs OS and PFS in AML patients more than the DA regimen does (Ding et al., 2024). These results emphasize the need for risk-adapted treatment strategies, particularly in non-intensive settings where 2022 ELN guidelines may not apply.

HSCT significantly improved survival, though its benefit was limited in *TP53*-mutated patients (He et al., 2024; Kim et al., 2024). Notably, HSCT failed to confer survival benefits in this subgroup, with all five patients undergoing transplantation dying post-transplant (Zhao et al., 2023). OS and PFS were the shortest for *TP53*, followed by *KIT*. The occurrence rate of the *WT1* gene in our study was 12.34%. *TP53* is mutually exclusive with *WT1*, while *CEBPA* is prone to coexist with *WT1*. The median OS of *WT1* was not reached, and the median PFS was 10.6 months. In addition, our study found that the *CEBPA* group indicated a good prognosis, but if combined with *WT1* gene mutation, the prognosis might be poor (Wang et al., 2022). *KIT* mutations, not included in ELN criteria, demonstrated prognostic significance comparable to *TP53*, necessitating their reevaluation in risk models. Interestingly, we found a strange phenomenon, 2022 ELN risk stratification is the same, but the actual prognosis is completely different. Statistical analysis of their baseline characteristics revealed no significant differences in Group A, except for LDH level and HSCT. In Group B, the only significant difference was PB%. Co-mutation analysis revealed distinct patterns—Group A (OS < 18 months) was enriched for *NPM1, FLT3, CEBPA* and *TET2* co-mutations, whereas Group B (OS > 18 months) exhibited frequent *TET2, ASXL1, WT1* and *CEBPA* associations. These findings highlight the inadequacy of current risk models in accounting for mutation interactions and suggest that 2022 ELN stratification may misclassify patients with specific co-mutation profiles. As proposed by Dohner et al. (2024b), a four-gene model (*TP53, FLT3-ITD, NRAS, KRAS*) demonstrates superior predictive accuracy for elderly AML patients receiving non-intensive therapy. Our study emphasizes the need for integrated models that incorporate both ELN criteria and mutation-specific signatures. These results advocate for a refined risk model integrating ELN criteria with mutation-specific signatures, particularly for elderly or non-intensive therapy patients. Future studies should validate the prognostic role of *KIT* and explore gene-environment interactions to optimize personalized care. The retrospective design and single-center cohort limit generalizability. Larger prospective studies are needed to validate prognostic role and optimize co-mutation scoring systems.

## Conclusions

In conclusion, AML prognosis is influenced by genetic and clinical factors, necessitating individualized risk stratification. Our study confirms that the 2022 ELN guidelines improve intermediate/adverse risk classification but underestimate variability within groups, particularly in patients with *FLT3, CEBPA*, or *TET2* mutations. *TP53* and *KIT* mutations were strongly associated with poor outcomes, warranting inclusion in prognostic models. HSCT significantly improved survival, though its benefit was limited in *TP53*-mutated patients. Our findings underscore the need for molecular data integration to refine AML risk stratification.

##  Supplemental Information

10.7717/peerj.20436/supp-1 Supplemental Information 1 Supplementary Information

10.7717/peerj.20436/supp-2 Supplemental Information 2 Raw data

## Additional Information and Declarations

### Data Availability

The following information was supplied regarding data availability: The raw data is available in the Supplemental File.
