# Supplementary material for: Retrospective study on the clinical outcomes and characteristics of acute myeloid leukemia: different outcomes in the same risk group
Source: PeerJ. 2025 Dec 5;13:e20436. doi: 10.7717/peerj.20436 (PMC12684409; doi:10.7717/peerj.20436)
Supplement: Supplemental Information 1 [file peerj-13-20436-s001.pdf]

Supplementary Information

Table S1.56 genes strongly associated with myeloid tumors.
Table S2. The genes according to functional classification.
Table S3. Baseline characteristics of AML patients across ELN 2022 risk groups.
Table S4. Distribution of genes within group A (OS < 18 months).
Table S5. Distribution of genes within group B (OS > 18 months).

Figure S1. Genetic variants landscape for patients with AML in the current study. Mutation types for each gene are colored using different colors, and each column represents an individual patient.
Figure S2. Risk distribution between the ELN−2017 and ELN-2022 risk stratification.
Figure S3. Univariable Cox proportional hazards models for OS.
Figure S4. Multivariate Cox proportional hazards models for OS.
Figure S5. Univariable Cox proportional hazards models for PFS.
Figure S6. Multivariate Cox proportional hazards models for PFS.

Table S1.56 genes strongly associated with myeloid tumors.

56 genes strongly associated with myeloid tumors							
ABL1	ARID2	ASXL1	ASXL2	BCOR	BCORL1	CALR	CBL
CDC25C	CEBPA	CSF3R	CSNK1A1	DDX41	DNMT3A	ETNK1	ETV6
EZH2	FLT3	GATA2	GNAS	GNB1	IDH1	IDH2	JAK2
JAK3	KDM6A	KIT	KRAS	MPL	NF1	NPM1	NRAS
PHF6	PIGA	PPM1D	PRPF8	PTPN11	RUNX1	SAMD9	SAMD9L
SBDS	SETBP1	SETD2	SF1	SF3B1	SH2B3	SRSF2	STAG2
STAT3	TET1	TET2	TP53	U2AF1	UBA1	WT1	ZRSR2

Table S2. The genes according to functional classification.

Functional cluster	Genes
Transcription factor genes	ARID2, CEBPA, ETV6, GATA2, GNAS, NPM1, PHF6, RUNX1
Signal transduction genes	ABL1, CALR, CALR, CSF3R, ETNK1, FLT3, GNB1, JAK2, JAK3, KIT, MPL, NF1, PPM1D, PTPN11, SETBP1
RNA splicing factor genes	DDX41, PRPF8, SF1, SF3B1, SRSF2, STAT3, U2AF1, ZRSR2
Proto-oncogene	CBL, KRAS, NRAS
Epigenetic regulation	ASXL1, ASXL2, BCOR, BCORL1, DNMT3A, EZH2, IDH1, IDH2, KDM6A, STAG2, TET1, TET2, WT1
Cell cycle, differentiation, and proliferation regulation genes	CDC25C, CSNK1A1, SAMD9, SAMD9L, SH2B3, UBA1
Cancer suppressor gene	SETD2, TP53
Others	SBDS

Table S3. Baseline characteristics of AML patients across ELN 2022 risk groups

Characteristics	2022ELN favorable and intermediate		p	2022ELN adverse		p
	OS > 18	OS < 18		OS > 18	OS < 18	
N	37	38		28	43	
Female (%)	22 (59.5)	19 (50.0)	0.555	17 (60.7)	22 (51.2)	0.585
Age < 60 (%)	33 (89.2)	29 (76.3)	0.243	19 (67.9)	25 (58.1)	0.566
HB < 70 (%)	20 (54.1)	13 (34.2)	0.134	9 (32.1)	16 (37.2)	0.855
PLT < 30(%)	15 (40.5)	19 (50.0)	0.555	17 (60.7)	15 (34.9)	0.058
Karyotype abnormal (%)	13 (35.1)	15 (39.5)	0.881	10 (35.7)	24 (55.8)	0.157
LDH abnormal (%)	21 (56.8)	37 (97.4)	**<0.001**	18 (64.3)	24 (55.8)	0.644
BM < 50 (%)	8 (21.6)	10 (26.3)	0.837	10 (35.7)	26 (60.5)	0.073
PB < 50(%)	16 (43.2)	15 (39.5)	0.923	12 (42.9)	35 (81.4)	**0.002**
Chemotherapy (%)	1 (2.7)	6 (15.8)	0.15	3 (10.7)	13 (30.2)	0.263
	21 (56.8)	21 (55.3)		15 (53.6)	17 (39.5)	
	10 (27.0)	5 (13.2)		4 (14.3)	4 (9.3)	
	5 (13.5)	6 (15.8)		6 (21.4)	9 (20.9)	
NO HSCT (%)	12 (32.4)	25 (65.8)	**0.008**	13 (46.4)	29 (67.4)	0.13

Table S4. Distribution of genes within group A (OS < 18 months).

group A (OS < 18 months) N=38	
Gene	N
ASXL2	1
NF1	1
RUNX1	1
SETD2	1
STAT3	1
TET1	1
No mutations	1
ARID2	2
CBL	2
GNAS	2
PHF6	2
PTPN11	2
SH2B3	2
CSF3R	3
JAK3	3
SETBP1	3
WT1	3
MPL	4
NRAS	4
KRAS	5
TET2	6
CEBPA	8
CEBPA+TET2+	4
KIT	8
NPM1	10
NPM1+FLT3+	10
DNMT3A	15
DNMT3A+NPM1+	10
DNMT3A+FLT3+	9
FLT3	15

Table S5. Distribution of genes within group B (OS > 18 months).

group B(OS > 18 months) N=28	
Gene	N
ASXL2	1
NF1	1
RUNX1	1
SETD2	1
STAT3	1
TET1	1
No mutations	1
ARID2	2
CBL	2
GNAS	2
PHF6	2
PTPN11	2
SH2B3	2
CSF3R	3
JAK3	3
SETBP1	3
WT1	3
MPL	4
NRAS	4
KRAS	5
TET2	6
CEBPA	8
CEBPA+TET2+	4
KIT	8
NPM1	10
NPM1+FLT3+	10
DNMT3A	15
DNMT3A+NPM1+	10
DNMT3A+FLT3+	9
FLT3	15

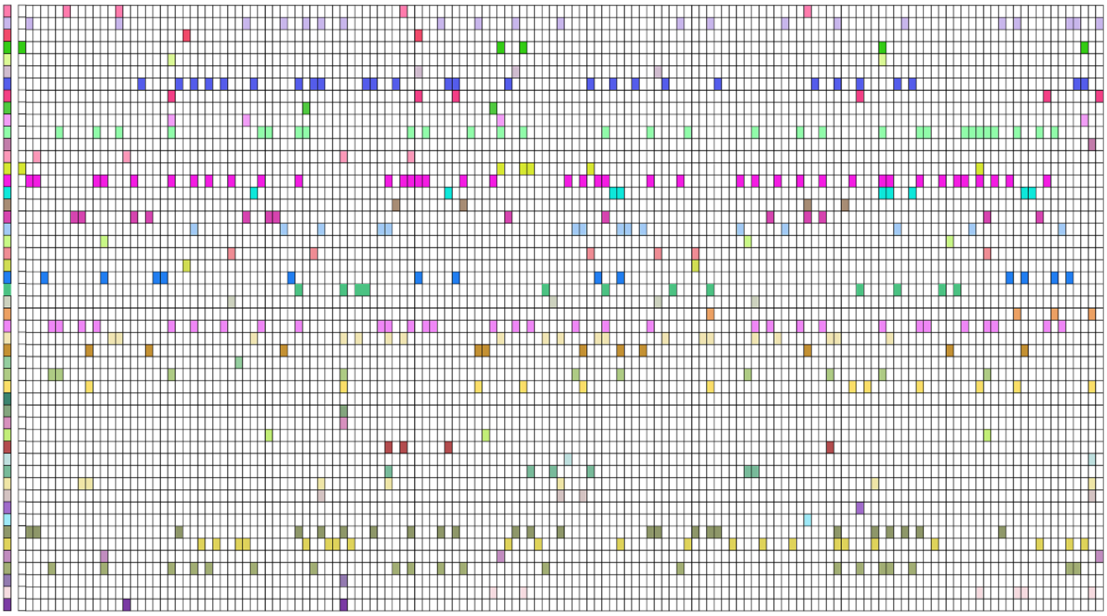

Figue S1. Genetic variants landscape for patients with AML in the current study. Mutation types for each gene are colored using different colors, and each column represents an individual patient.

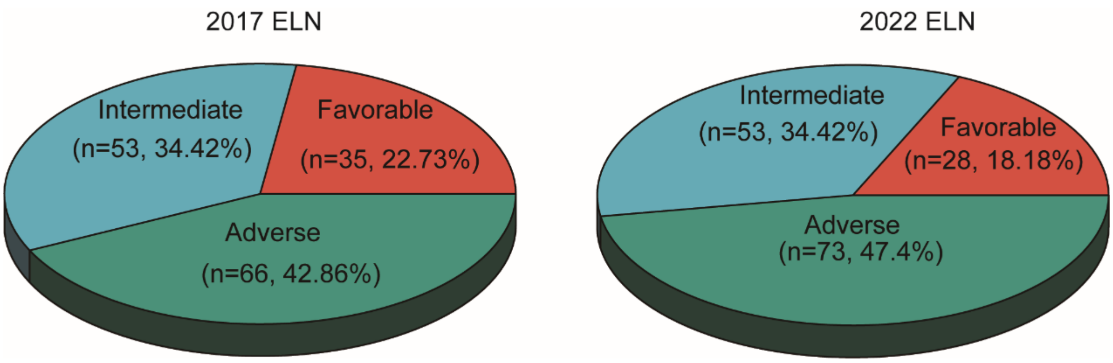

Figure S2. Risk distribution between the ELN−2017 and ELN-2022 risk stratification.

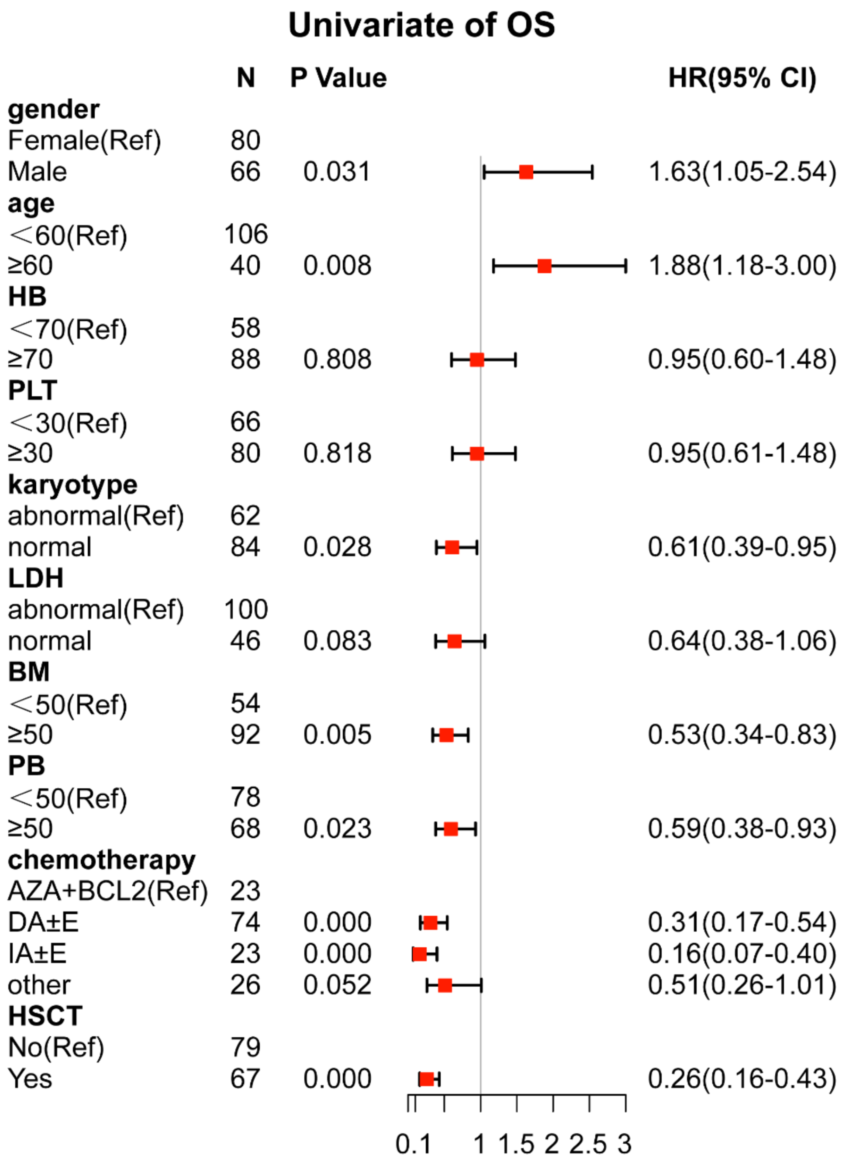

Figure S3. Univariable Cox proportional hazards models for OS.

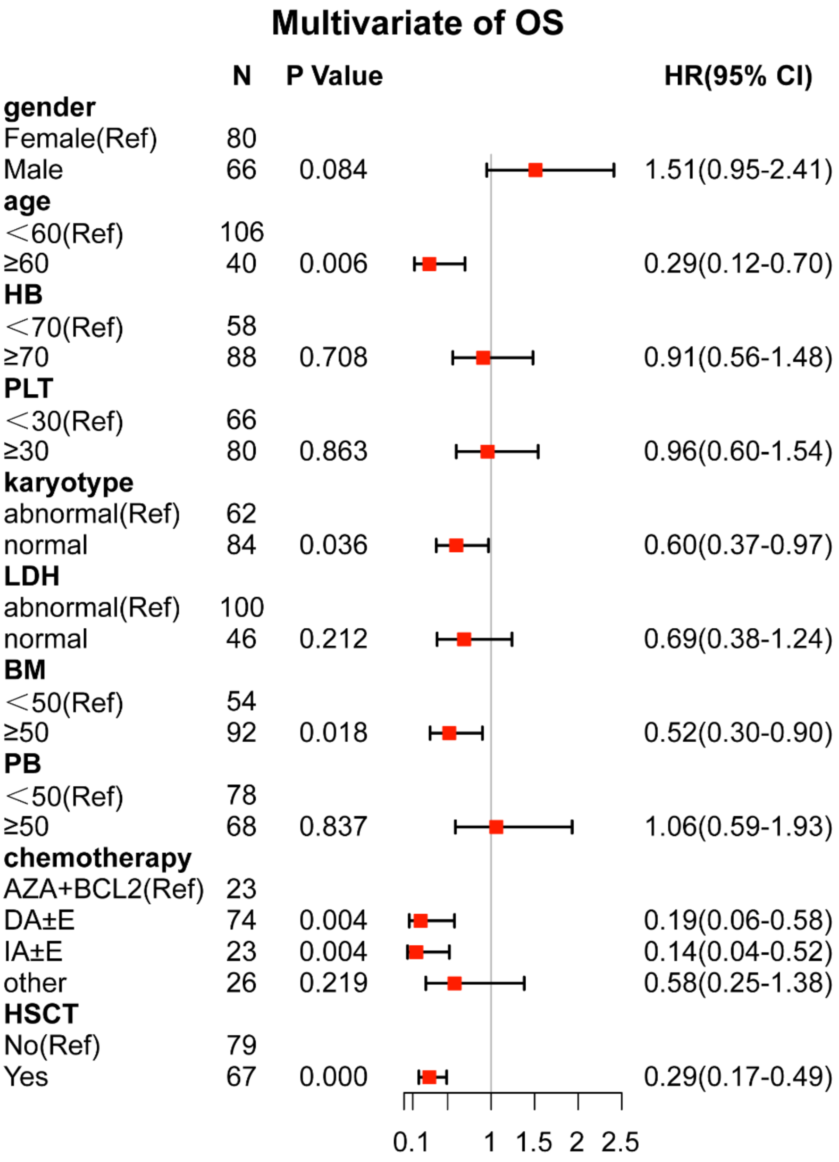

Figure S4. Multivariate Cox proportional hazards models for OS.

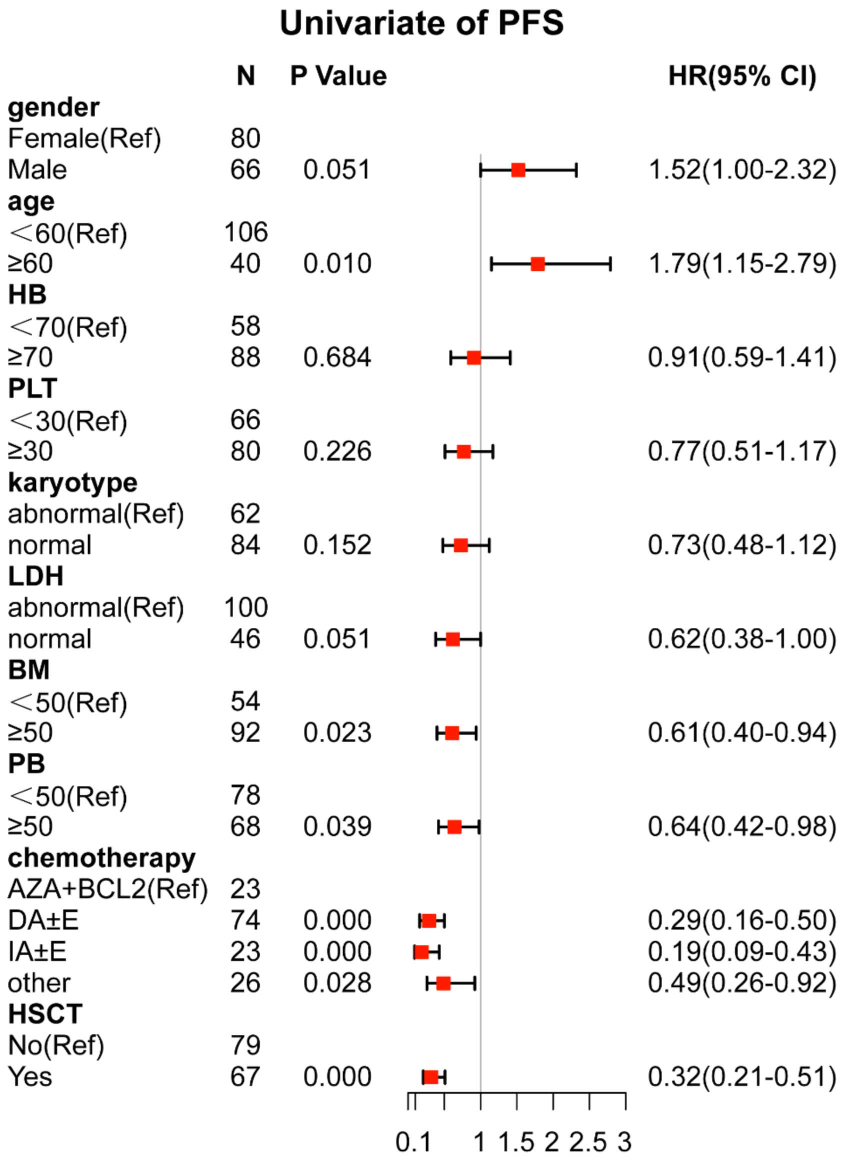

Figure S5. Univariable Cox proportional hazards models for PFS.

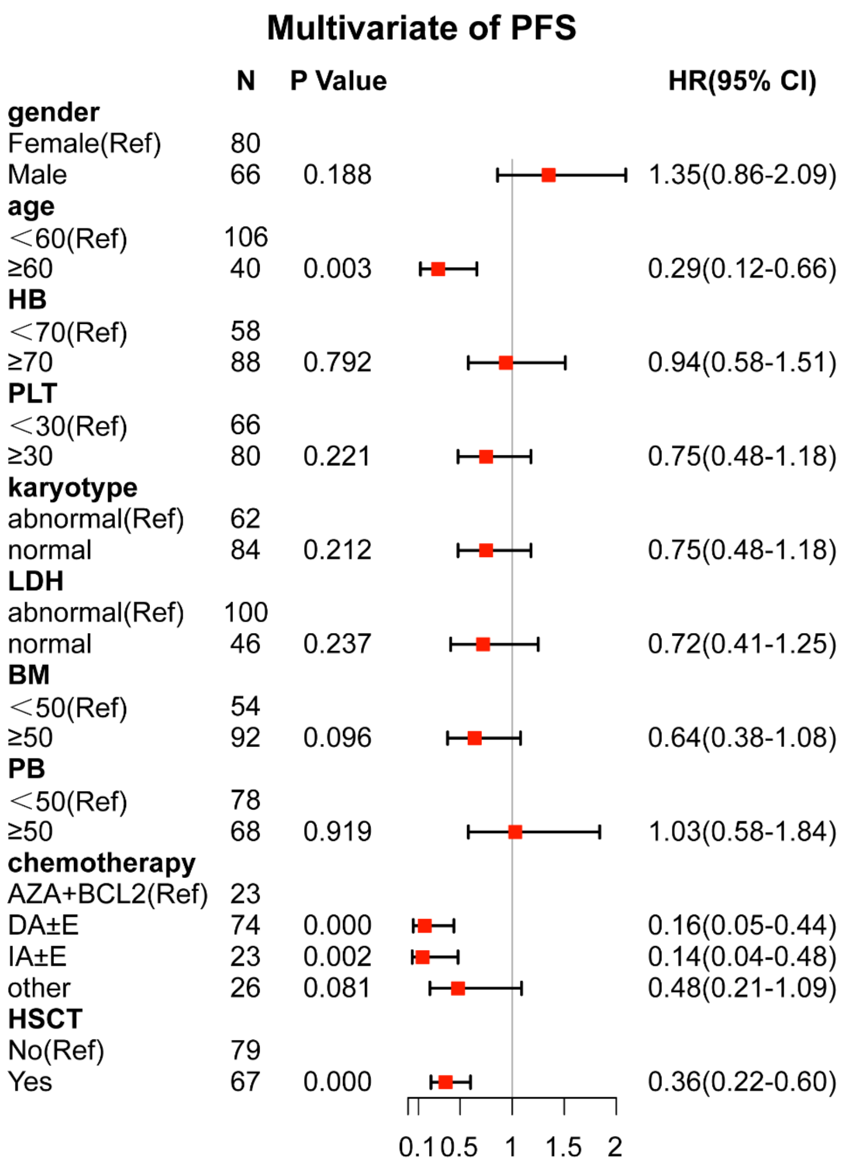

Figure S6. Multivariate Cox proportional hazards models for PFS.
